# The Role of Obesity Training in Medical School and Residency on Bariatric Surgery Knowledge in Primary Care Physicians

**DOI:** 10.1155/2015/841249

**Published:** 2015-08-03

**Authors:** Fatima Cody Stanford, Erica D. Johnson, Mechelle D. Claridy, Rebecca L. Earle, Lee M. Kaplan

**Affiliations:** ^1^Obesity, Metabolism and Nutrition Institute, Gastrointestinal Unit and MGH Weight Center, Massachusetts General Hospital, Boston, MA 02114, USA; ^2^Harvard Medical School, Boston, MA 02115, USA; ^3^Harvard Kennedy School of Government, Cambridge, MA 02138, USA; ^4^University of Pittsburgh School of Medicine, Pittsburgh, PA 15261, USA; ^5^Department of Community Health and Preventive Medicine, Morehouse School of Medicine, Atlanta, GA 30310, USA

## Abstract

*Objective*. US primary care physicians are inadequately educated on how to provide obesity treatment. We sought to assess physician training in obesity and to characterize the perceptions, beliefs, knowledge, and treatment patterns of primary care physicians. *Methods*. We administered a cross-sectional web-based survey from July to October 2014 to adult primary care physicians in practices affiliated with the Massachusetts General Hospital (MGH). We evaluated survey respondent demographics, personal health habits, obesity training, knowledge of bariatric surgery care, perceptions, attitudes, and beliefs regarding the etiology of obesity and treatment strategies. *Results*. Younger primary care physicians (age 20–39) were more likely to have received some obesity training than those aged 40–49 (OR: 0.08, 95% CI: 0.008–0.822) or those 50+ (OR: 0.03, 95% CI: 0.004–0.321). Physicians who were young, had obesity, or received obesity education in medical school or postgraduate training were more likely to answer bariatric surgery knowledge questions correctly. *Conclusions*. There is a need for educational programs to improve physician knowledge and competency in treating patients with obesity. Obesity is a complex chronic disease, and it is important for clinicians to be equipped with the knowledge of the multiple treatment modalities that may be considered to help their patients achieve a healthy weight.

## 1. Introduction

Obesity has reached epidemic proportions in the United States, with recent data indicating that more than 78 million adults (36%) and 12.5 million children and adolescents (17%) in the United States have this disorder [1, 2]. The enormous health and economic tolls of obesity and its profound adverse effects on quality of life underscore the need for both increased prevention and effective treatment [3, 4]. Obesity has been linked to more than 65 comorbidities, including coronary artery disease, type 2 diabetes mellitus, hypertension, hypercholesterolemia, more than a dozen cancers, and a host of other disease processes [5]. Although obesity has a substantial impact on patient care in all disciplines in medicine, physician and medical student education about obesity is limited [6, 7]. These educational gaps may leave physicians inadequately prepared to address the needs of this substantial and growing portion of their patient population.

Previous studies of physician treatment of obesity demonstrate that there is limited physician-directed care in obesity. Many physicians fail to recognize obesity as a chronic disease. Others recognize obesity as a disease but choose not to address obesity during their limited time with patients [8–11]. Few studies, however, have assessed the root cause of physician perceptions about obesity and its treatment. Attitudes about healthcare in general and obesity in particular are often shaped during medical school and residency training [12]. In order to adequately train physicians to tackle obesity, they need a rigorous background in the biologic and pathophysiological foundations of obesity [13]. Evidence strongly suggests that primary care providers lack confidence in their knowledge and skills in providing obesity care [14]. In our study, we sought to determine whether obesity training correlated with knowledge, beliefs, and attitudes about the etiology of and treatment of patients with obesity. We therefore hypothesize that primary care physicians who have undergone obesity training will be more likely to answer questions correctly regarding care for patients with obesity and to consider multiple modalities of treatment to help patients achieve durable control of their obesity and its sequelae [15, 16]. Among the many important targets for physician education about obesity are better understanding of its physiologic basis, complexity, and heterogeneity; diminishing negative attitudes towards patients with obesity; mitigation of obesity bias; and recognition that even modest weight reduction can lead to a substantial improvement or prevention of comorbidities which appear to be particularly important [17–20].

## 2. Materials and Methods

### 2.1. Participants and Data Collection

We established an online survey for adult primary care physicians in practices affiliated with the Massachusetts General Hospital (MGH). Within Partners HealthCare, MGH provides primary care to more than 200,000 patients in 15 locations in Greater Boston on an annual basis. We surveyed internal medicine, internal medicine/pediatrics, and family medicine physicians through the Partners HealthCare Research Electronic Data Capture (REDCap), a secure, web-based application designed to support data capture for research studies [21]. The period of data collection was 3 months (July 2014–October 2014). Of the 237 surveys sent to MGH primary care physicians via REDCap, 76 survey participants (32%) completed the survey. Six survey participants did not provide electronic consent to release their survey results. Therefore, only 70 survey participants were used for this analysis. Participants who completed the survey were offered educational materials from the 2014 Harvard Medical School Course in Obesity Medicine [22]. The Institutional Review Board of the Massachusetts General Hospital approved this study.

### 2.2. Survey Instrument

We developed an anonymous cross-sectional survey to obtain self-reported information. We obtained demographics including (a) primary medical specialty, (b) gender, (c) degree type, (d) racial/ethnic background, (e) age, (f) weight, (g) height, (h) personal history of overweight or obesity, (i) year of medical school graduation, (j) county of birth, and (k) personal history of chronic diseases. In addition to demographics, we obtained information on primary care physician's personal health habits (i.e., frequency of disclosure of nutrition, physical activity, stress reduction methods, and use of smartphone application to manage weight), obesity training (i.e., duration and type of obesity training: lecture, online training, group discussion, hospital rotation, and special topic course), frequency of recording a diagnosis of overweight or obesity in the electronic medical record, knowledge of bariatric surgery care, perceptions, attitudes, and beliefs regarding obesity treatment.

### 2.3. Statistical Analysis

We used descriptive statistics to characterize our sample according to whether or not physicians received obesity related training. We calculated mean body mass index (BMI) for each training group, and we characterized obesity training into two categorical variables: no obesity training and some obesity training. No obesity training was defined as those who received less than one hour of training of any type (e.g., lecture, online training, group discussion, hospital rotation, and special topic course). Chi-square analyses and Fisher's exact test were used to evaluate differences in proportions for those who received obesity training and those who did not receive obesity training.

In addition, we conducted multivariable logistic regression models and classified those who received some obesity training and those who were confident in their ability to treat obesity as the outcome. Gender, age, and BMI were used as explanatory predictor variables for those who received some obesity training and for those who were confident in their ability to treat obesity. To characterize differences in those who received obesity training, we conducted an additional multivariable model, which examined the association between age and physicians' confidence in treating obesity and receiving obesity training (outcome) or receiving no obesity training (outcome). All analyses were performed using SAS software, version 9.2.

## 3. Results

Table 1 characterizes our overall sample stratified by obesity training. 59% of physician survey respondents (41 physicians) received at least one hour of obesity training. In both categories (no obesity training versus some obesity training), adult primary care physician survey respondents were most likely to have the following demographics: normal BMI, female, Caucasian, no chronic disease processes, and completion of an internal medicine residency. Younger physician respondents, those aged 20–39, were more likely to have received some obesity training compared to physicians 40 and older.  Table 2 characterizes physician's perceptions of obesity by obesity training. While these results were not statistically significant, physicians with obesity training were more likely to believe that obesity is a chronic disease process, refer appropriate patients for bariatric surgery evaluation, and feel that bariatric surgery is a safe and useful tool for appropriate patients.  Table 3 characterizes physician respondents' knowledge about bariatric surgery care by obesity training. Physicians with obesity training were significantly more likely to answer bariatric surgery knowledge questions correctly when compared with those without training. In Table 4, age, specifically being in the 20–39 age group, was significantly associated (*P* < 0.05) with the likelihood that a physician survey respondent received some obesity training. In Table 5, younger physicians (age 20–39), physicians with normal weight, and those with some obesity training were more likely to feel confident in treating obesity. In Table 6, persons who did not receive any obesity training appear more confident in their ability to treat obesity, but this result was not statistically significant.

**Figure 1:**
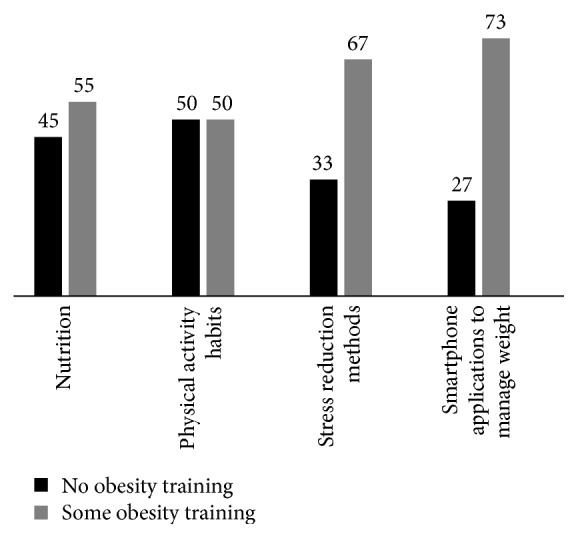
Physicians that almost always/frequently disclose their own healthy habits to influence patient behavior.

As noted in Figure 1, physicians with some obesity training were more likely to disclose their own personal habits to influence the behavior of their patients. Physicians with some obesity training were most likely to consider the following to be major barriers to evaluating and/or managing patients with overweight and obesity in their practice: (1) not having enough time, (2) not being part of my professional role, (3) inadequate training, (4) fear of offending the patient, (5) too difficult for patients to change, (6) lack of effective tools and information to give to patients, and (7) long wait times for referrals to obesity medicine specialists (Supplemental Figure 1 in Supplementary Material available online at http://dx.doi.org/10.1155/2015/841249). Alternatively, physicians with no obesity training were most likely to consider the following to be major barriers to evaluating and/or managing patients with overweight and obesity in their practice: (1) inadequate reimbursement, (2) lack of adequate referral services for diet, physical activity, and weight, (3) patients being generally not interested in improving their weight status, and (4) lack of effective treatment options. Across all questions in which physician survey respondents were given a scenario to consider various treatment modalities for patient with obesity, physicians with some obesity training were more likely to consider all possible treatment options to help a patient lose weight (self-direction, allied health, and physician directed therapy, weight loss medications, and bariatric surgery) than those with no obesity training (Supplemental Figures 2–4).

## 4. Discussion

In June 2013, the American Medical Association (AMA) voted to acknowledge obesity as a chronic disease, and it has promoted a robust discussion in the medical community about the range of interventions which should be utilized to prevent and treat obesity [23]. Bleich and colleagues evaluated physician practice patterns of obesity diagnosis and weight-related counseling in a national cross-sectional study [24]. They found that primary care physicians overwhelmingly supported additional training in obesity, and these physicians felt that nutritionists/dietitians were most qualified to provide obesity care. They concluded that there is a need for improved medical education related to obesity. Jay and colleagues evaluated internal medicine, pediatrics, and psychiatry physicians from an academic institution, and they determined that physician attitudes towards obesity are associated with competency (more competency with better attitudes), specialty (pediatrics specialists with more positive attitudes than psychiatry and internal medicine specialists), and years since postgraduate training (those with fewer years since postgraduate training with more positive attitudes) [25].

Unfortunately, a majority of patients with obesity do not receive any weight loss advice from health professionals, and when they do receive advice it is usually restricted to information on calorie reduction and increased physical activity [26, 27]. Physicians should investigate other factors which might contribute to increased weight such as poor sleep quality and duration, circadian rhythm disturbances, and use of weight promoting medications. In addition, studies have shown that rates of weight counseling by primary care physicians have declined despite increased rates of overweight and obesity in the United States [26, 28]. In a review conducted by Teixeira and colleagues of thirteen studies between 1991 and 2011 in which they synthesized the main investigation results regarding beliefs, attitudes, and practices of healthcare providers, they found that lack of appropriate understanding and adequate competence regarding obesity likely contributes to ambivalent belief development and negative attitudes toward obese individuals, who are described as unmotivated, lazy, and lacking self-control [29]. Physicians with greater knowledge, more positive attitudes toward obesity management, and access to more resources are more likely to provide weight management in primary care settings [30].

One important barrier that impedes optimal care delivery to patients with obesity is weight bias and stigma. In a national sample of over 4700 medical students, Phelan and colleagues evaluated implicit and explicit weight bias. They determined that a majority of students exhibited implicit (74%) and explicit (67%) weight bias [18]. In another study of postgraduate health discipline students, Puhl and colleagues reported that patients with obesity are a common target of negative attitudes and derogatory humor by peers (63%), healthcare providers (65%), and instructors (40%) [20]. It must be noted that physicians themselves also experience weight bias from patients as studies have demonstrated that providers perceived to be overweight or obese are vulnerable to biased attitudes from patients and that providers' excess weight negatively affects patients' perceptions of their credibility, level of trust, and inclination to follow medical advice [19]. In this cross-sectional web-based survey, we assessed primary care physician knowledge and training in obesity, obesity treatment and referrals patterns, and attitudes and beliefs about obesity. We found that there were generational differences in obesity education. Younger physicians (aged 20–39) were most likely to have received some obesity training. Physician survey respondents who were young (age 20–39), had obesity (BMI ≥ 30 kg/m^2^), and had some obesity training (>1 hour) were significantly more likely to answer bariatric survey knowledge questions correctly compared to their peers. Further, obesity education impacted both knowledge of treatment options and referral patterns. Physicians with obesity training were more likely to consider obesity a chronic disease process. They were also more likely to consider multiple modalities to treat patients who have obesity.

The results of our study should be interpreted in the context of its limitations. While our survey ascertained several aspects of obesity and perceptions, knowledge, and attitudes of our physician survey respondents, it is difficult to generalize our results to all primary care physicians. Our study was conducted at a large academic medical center in which there are practitioners with less racial/ethnic and medical training diversity than other sites which indicates that survey respondents are not likely representative of physicians throughout the United States. Additionally, our survey respondents have access to a large tertiary care referral center in which obesity medicine physicians, psychologists, and dietitians provide care to patients whom they have difficulty helping to achieve a healthy weight. There was likely selection bias in the survey respondents with regard to physicians who have an interest in obesity, and this bias likely contributed to a lower than expected response rate to the survey. Finally, many physician survey respondents did not receive more than one hour of obesity education in their entire postgraduate training. It is challenging to decipher whether this small amount of training can be correlated with better patient outcomes.

## 5. Conclusions

Our current study supports previous studies which show that physicians do not receive adequate training in obesity. Survey responses indicate that obesity training increases physician knowledge of obesity treatment and may improve treatment rates and diversify treatment options for patients with obesity. Given the high prevalence of obesity and its role in causing, exacerbating, or complicating many other chronic diseases, there appears to be an important need for educational programs focused on improving resident and physician knowledge about obesity and comorbidities. Physicians should be knowledgeable about the impact of obesity in their patient population and be adequately prepared to assess obesity and administer care to patients with obesity in inpatient and outpatient settings. Finally, they should strive to eliminate their implicit and explicit weight bias towards patients with obesity so they are able to provide unparalleled care to this growing segment of the population.

## Supplementary Material

The supplemental material provides survey results on barriers to providing obesity care in primary care practices (Supplemental Figure 1) and the range of treatment modalities primary care physicians would be likely to employ in patients who meet medical criteria for bariatric surgery (Supplemental Figures 2-4) by obesity training status.

## Figures and Tables

**Table 1:** Characteristics of study sample by training.

	No obesity related training (*n* = 29; 41%)	Obesity related training (*n* = 41; 59%)
Mean BMI (kg/m^2^)	24.74	25.73
Sex		
Male	9 (36)	16 (64)
Female	20 (44)	25 (56)
Age		
20–39	**1 (6)**	**16 (94)**
40–49	**8 (42)**	**11 (58)**
50+	**20 (59)**	**14 (41)**
Body mass index		
Underweight/normal weight	19 (49)	20 (51)
Overweight	5 (26)	14 (74)
Obese	5 (42)	7 (58)
Ethnic background		
Asian/Pacific Islander	3 (38)	5 (62)
Caucasian (non-Hispanic)	25 (43)	33 (57)
Hispanic	1 (50)	1 (50)
Other	0 (0)	2 (100)
Chronic diseases		
Asthma	5 (42)	7 (58)
Dyslipidemia	1 (33)	2 (67)
Gastroesophageal reflux disease	1 (50)	1 (50)
Coronary artery disease	0 (0)	1 (100)
Hypertension	4 (57)	3 (43)
Obstructive sleep apnea	0 (0)	1 (100)
Osteoarthritis	0 (0)	1 (100)
Polycystic ovarian syndrome	0 (0)	1 (100)
Depression	1 (13)	7 (87)
None	17 (50)	17 (50)
Medical specialty		
Internal medicine	24 (43)	32 (57)
Family medicine	1 (50)	1 (50)
Internal medicine/pediatrics	2 (22)	7 (78)
Other	2 (67)	1 (33)

Results delineated in bold indicate statistically significant differences across the variable at *P* < 0.05. We used Chi-square and Fisher's exact tests to determine differences in proportions between those that had no obesity related training and those who had some obesity related training.

**Table 2:** Physician perceptions of obesity by training.

	No obesity related training (*n* = 29; 41%)	Obesity related training (*n* = 41; 59%)
Obesity is a chronic disease		
Strongly agree/agree	29 (43)	38 (57)
Neutral	0 (0)	2 (100)
Strongly disagree/disagree	0 (0)	1 (100)
I am generally successful in treating patients for obesity		
Strongly agree/agree	4 (44)	5 (56)
Neutral	7 (44)	9 (56)
Strongly disagree/disagree	18 (40)	27 (60)
I would treat obesity more regularly if there was reimbursement set aside for that purpose		
Strongly agree/agree	9 (56)	7 (44)
Neutral	11 (35)	20 (65)
Strongly disagree/disagree	9 (39)	14 (61)
If a patient meets the standard criteria for bariatric surgery, I would recommend evaluation by a bariatric surgeon		
Strongly agree/agree	28 (43)	37 (57)
Neutral	1 (25)	3 (75)
Strongly disagree/disagree	0 (0)	1 (100)
I feel bariatric surgery is a safe option for treating obesity		
Strongly agree/agree	22 (41)	31 (58)
Neutral	7 (47)	8 (53)
Strongly disagree/disagree	0 (0)	2 (100)
I feel bariatric surgery is a useful tool for treating obesity		
Strongly agree/agree	29 (43)	38 (57)
Neutral	0 (0)	3 (100)
Strongly disagree/disagree	0 (0)	0 (0)

Results indicate statistically significant differences across the variable at *P* < 0.05. We used Chi-square and Fisher's exact tests to determine differences in proportions between those that had no obesity related training and those who had some obesity related training.

**Table 3:** Physician knowledge of bariatric surgery by training.

	No obesity related training (*n* = 29; 41%)	Obesity related training (*n* = 41; 59%)
Which BMI would typically qualify a patient for bariatric surgery? (35+ with comorbidities)		
Correct	15 (36)	27 (64)
Incorrect	14 (50)	14 (50)
The average expected excess body weight loss from Roux-en-Y gastric bypass is 50–75%?		
Correct	4 (27)	11 (73)
Incorrect	25 (45)	30 (55)
The national 30-day mortality rate of patients who undergo Roux-en-Y gastric bypass is <1%?		
Correct	12 (32)	25 (68)
Incorrect	17 (52)	16 (48)
Patients who undergo bariatric surgery are expected to achieve their maximum weight loss within which of the following time frames? (12–18 months)		
Correct	20 (43)	26 (57)
Incorrect	9 (38)	15 (62)
Knowledge questions overall		
More than 50% correct	**28 (46)**	**33 (54)**
Less than 50% correct	**1 (11)**	**8 (89)**

Results delineated in bold indicate statistically significant differences across the variable at *P* < 0.05. We used Chi-square and Fisher's exact tests to determine differences in proportions between those that had no obesity related training and those who had some obesity related training.

**Table 4:** Factors associated with the likelihood that physicians received some obesity training.

	Odds ratio	95% CI interval	*P* value
Sex			
Female	1.00	Ref.	
Male	0.703	(0.257, 1.923)	0.4927
Age			
20–39	**1.00**	**Ref.**	
40–49	**0.08**	** (0.008, 0.822)**	**0.0300**
50+	**0.03**	** (0.004, 0.321)**	**0.0040**
Body mass index			
Underweight/normal weight	1.00	Ref.	
Overweight	0.376	(0.113, 1.247)	0.1097
Obese	0.752	(0.203, 2.782)	0.6692

Results delineated in bold indicate statistically significant differences across the variable at *P* < 0.05.

**Table 5:** Factors associated with a physician's confidence in treating obesity.

	Odds ratio	95% CI interval	*P* value
Sex			
Female	1.00	Ref.	
Male	0.761	(0.238, 2.435)	0.2126
Age			
20–39	1.00	Ref.	
40–49	**0.179**	** (0.037, 0.864)**	**0.0322**
50+	**1.049**	** (0.221, 4.982)**	**0.0036**
Body mass index			
Underweight/normal weight	1.00	Ref.	
Overweight	**0.977**	** (0.227, 4.198)**	**0.0010**
Obese	2.231	(0.583, 8.540)	0.2415
Training			
No training	1.00		
Some training	**1.083**	**(0.322, 3.642)**	**0.0164**

Results delineated in bold indicate statistically significant differences across the variable at *P* < 0.05.

**Table 6:** Obesity training status and correlation to confidence in obesity care.

	Odds ratio (95% CI) No training	Odds ratio (95% CI) Some training	*P* value
Age			
20–39	1.00	Ref	
40–49	**10.987 (1.147, 105.234)**	** 0.091 (0.010, 0.872)**	**0.0376**
50+	**22.797 (2.702, 192.324)**	**0.044 (0.005, 0.370)**	**0.0041**
Confidence			
Not confident in successfully treating obesity	1.00		
Confident in successfully treating obesity	1.158 (0.361, 3.714)	0.863 (0.269, 2.768)	0.0613

Results delineated in bold indicate statistically significant differences across the variable at *P* < 0.05.
