# Supplementary figures and images for: The Role of Obesity Training in Medical School and Residency on Bariatric Surgery Knowledge in Primary Care Physicians
Source: Int J Family Med. 2015 Aug 3;2015:841249. doi: 10.1155/2015/841249 (PMC4539067; doi:10.1155/2015/841249)

Supplemental Figure 1

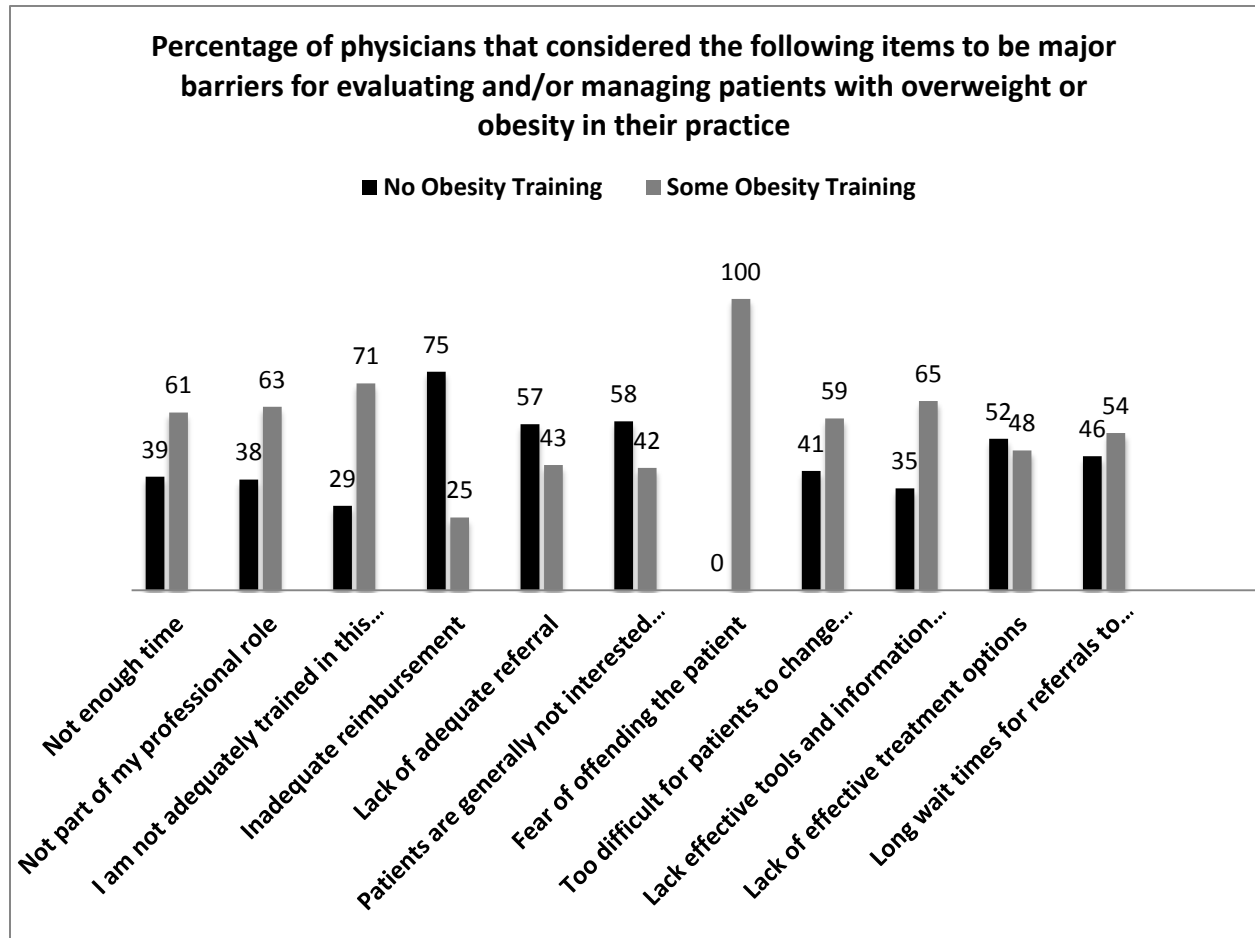

**Supplemental Figure 2**

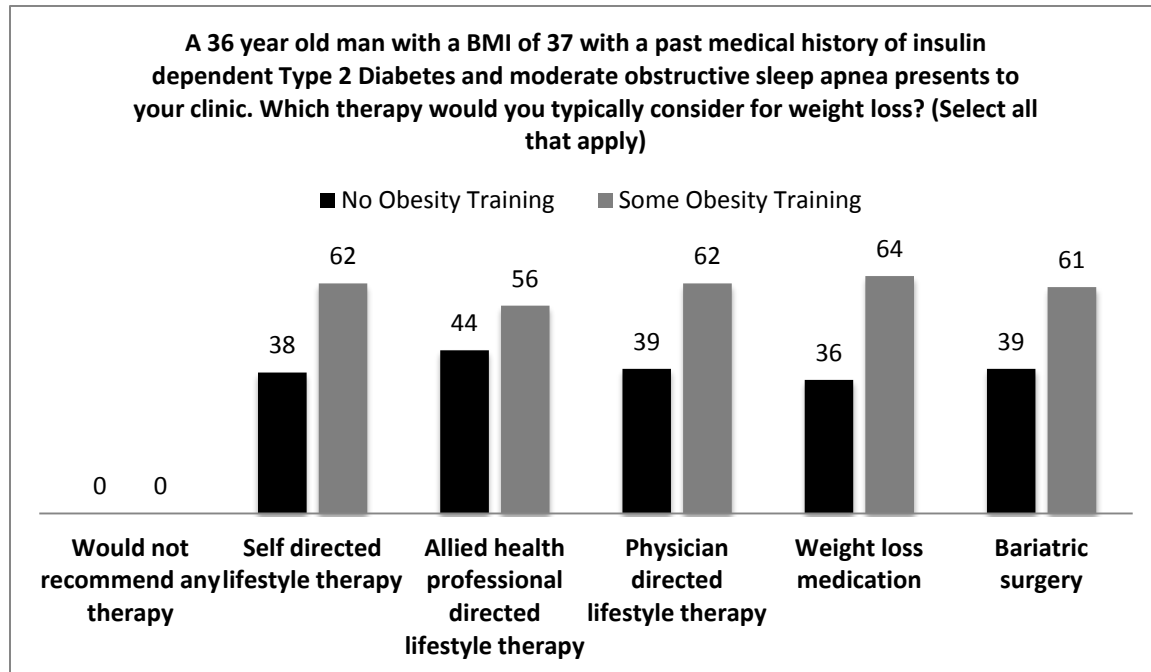

**Supplemental Figure 3**

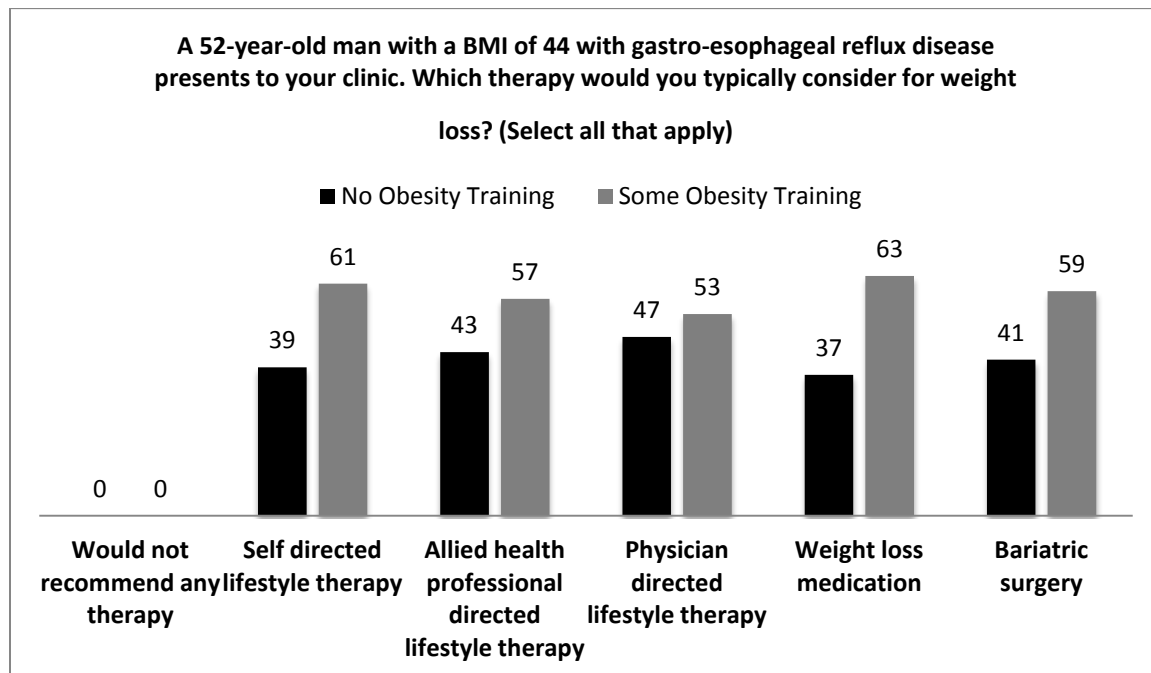

**Supplemental Figure 4**

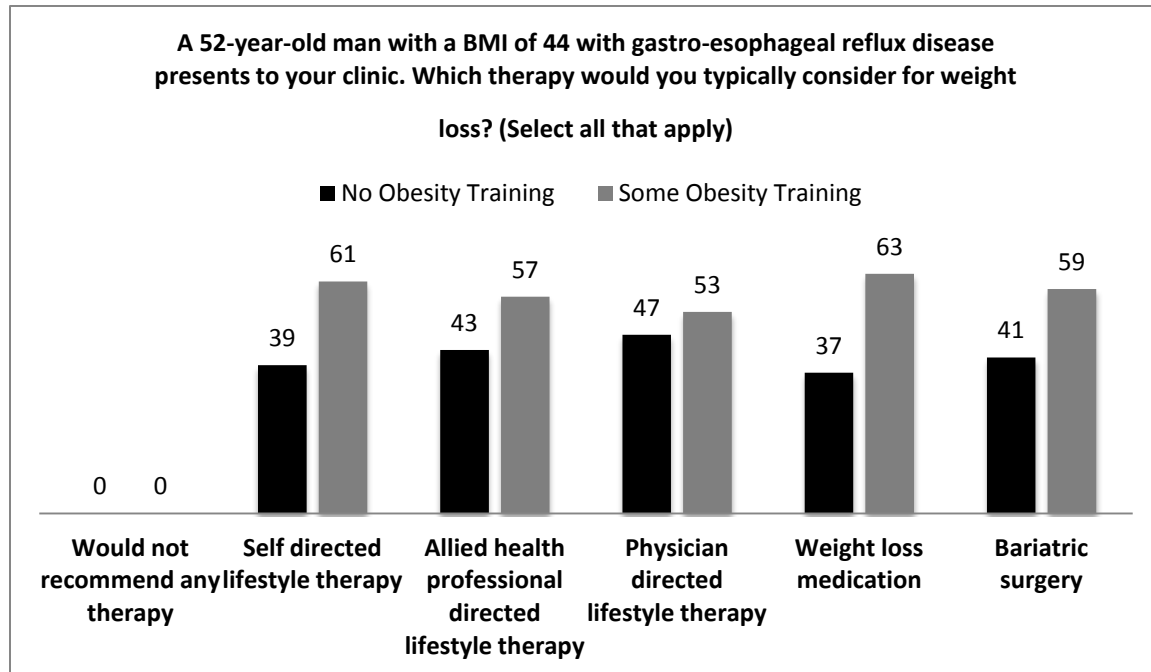

Supplement: Supplementary file 1 — The supplemental material provides survey results on barriers to providing obesity care in primary care practices (Supplemental Figure 1) and the range of treatment modalities primary care physicians would be likely to employ in patients who meet medical criteria for bariatric surgery (Supplemental Figures 2-4) by obesity training status. [file 841249.f1.pdf]
